# Extracellular matrix and dermal nerve growth factor dysregulation in prurigo nodularis compared to atopic dermatitis

**DOI:** 10.3389/fmed.2022.1022889

**Published:** 2022-12-21

**Authors:** Junwen Deng, Varsha Parthasarathy, Melika Marani, Zachary Bordeaux, Kevin Lee, Chi Trinh, Hannah L. Cornman, Anusha Kambala, Thomas Pritchard, Shihua Chen, Nishadh Sutaria, Olusola O. Oladipo, Madan M. Kwatra, Martin P. Alphonse, Shawn G. Kwatra

**Affiliations:** ^1^Department of Dermatology, Johns Hopkins University School of Medicine, Baltimore, MD, United States; ^2^Department of Anesthesiology, Duke University School of Medicine, Durham, NC, United States

**Keywords:** prurigo nodularis, atopic dermatitis, pruritus, transcriptome, immune, nerve growth factor, neuropathic

## Abstract

Prurigo nodularis (PN) is a chronic, pruritic, inflammatory skin disease characterized by hyperkeratotic nodules on the trunk and extremities. While there is growing research on the immunological basis of PN, the neuropathic and structural components of PN lesions are unknown. This study examines the inflammatory, neuropathic, and structural pathways in PN compared to atopic dermatitis (AD) using RNA-sequencing of the lesional and non-lesional skin tissue of PN and AD patients, as well as immunohistochemistry analysis of nerve growth factor (NGF), a neurotrophic factor that regulates nerve development. Transcriptomic analysis of skin biopsies revealed that compared to lesional AD skin, lesional PN skin had significantly increased expression of NGF, matrix metalloproteinases, OSM, MCEMP1, IL1α, IL1β, CXCL2, CXCL5, CXCL8, and insulin-like growth factors in PN compared to AD, and decreased expression of CCL13, CCL26, EPHB1, and collagens (COL4/6). Gene set enrichment analysis demonstrated higher enrichment of keratinization, cornified envelope, myelin sheath, TGF-beta signaling, extracellular matrix disassembly, metalloendopeptidase activity, and neurotrophin-TRK receptor signaling pathways in PN. On immunohistochemistry, PN lesions demonstrated higher dermal NGF expression compared to AD. We present novel findings demonstrating increased neurotrophic and extracellular matrix remodeling signatures in PN compared to AD, possibly explaining the morphological differences in their lesions. These signatures may therefore be important components of the PN pathogenesis and may serve as therapeutic targets.

## Introduction

Prurigo nodularis (PN) is a chronic, inflammatory skin disease characterized by hyperkeratotic and pruritic nodules on the extensor surfaces and trunk (1). PN patients often suffer from significant impairment to quality of life and have substantial comorbidity burden, including higher risks of having diabetes, cardiovascular disease, infectious disease, and neuropsychiatric diseases (2). Furthermore, PN is a particularly challenging skin condition to treat, for there are currently no FDA-approved therapies (1). Therefore, identification of disease biomarkers can aid in the future management of PN.

Though PN features both inflammatory and neuropathic dysregulation, the exact pathogenesis of PN is not yet well described. PN shares several inflammatory features with atopic dermatitis (AD), including cutaneous upregulation of interleukin (IL)-4R and Th22 transcriptomic signatures (3, 4). However, while PN may be associated with other inflammatory skin conditions such as AD, many patients present with PN alone or with other dermatologic findings, demonstrating the distinct etiologies of the two diseases.

In particular, the role of nerve growth factor (NGF), a neurotrophic growth factor which regulates nerve development, has not been previously examined in PN in relation to AD. Furthermore, chronic inflammation in skin diseases has been linked to extracellular modulators such as matrix metalloproteinases (MMPs), which have a pathological role in enabling immune cells to enter and exit inflamed skin (5). However, the dysregulation of MMPs in PN pathogenesis has received little attention. Therefore, we hypothesized that direct comparison of the cutaneous transcriptomes and immunohistochemical distribution of NGF in PN and AD patients would provide insight into the unique inflammatory and neuropathic mechanisms of PN.

## Methods

RNA sequencing was performed on skin punch biopsies from lesional and non-lesional areas of PN and AD patients with moderate-to-severe pruritus, in order to directly compare specific neuroimmune differences between the two conditions. Lesional and non-lesional skin biopsies were collected from 13 PN patients and 6 AD patients, as well as the healthy skin of 19 controls matched by age (±10 years), sex (male, female), and race (African American, White, and other race), using the same methodology previously employed by our group (3, 4). Lesional biopsies were collected from the most pruritic nodules, and non-lesional biopsies were collected from normal skin at least 10 cm from the lesion biopsy. Total RNA was extracted from skin tissue, and the KAPA Stranded mRNA-Seq Kit (Roche) was used to prepare RNA-seq libraries. RNA sequencing was performed on the Illumina NovaSeq 6000 sequencer (Illumina) and the data was processed and trimmed using the fastp toolkit (6). Reads were mapped to the GRCh38v93 version of the human transcriptome using the STAR RNA-seq alignment tool (7) and the resulting gene counts were compiled using the FeatureCounts tool (8). Data normalization and differentially expressed gene (DEG) calculations were conducted using *DESeq2* (Bioconductor). DEGs were defined as genes with a log_2_-fold change <−1.5 or >1.5. The false discovery rate (FDR) was calculated to control for multiple hypothesis testing. Pathway-level comparisons were performed using gene set enrichment analysis (GSEA) (9).

Immunohistochemistry (IHC) staining for NGF was performed on formalin-fixed, paraffin embedded sections on PN and AD samples (*n* = 8 each) and non-lesional samples (*n* = 3 each) matched by age (±10 years), sex (male, female), and race (African American, White, and other race). Epitope retrieval was performed using Ventana Ultra CC1 buffer (catalog# 6414575001, Roche). Anti-NGF (1:500 dilution; catalog# ab52918, Abcam) primary antibody was applied and detected using an anti-rabbit HQ detection system (catalog# 7017936001 and 7017812001, Roche) followed by ChromoMap DAB IHC detection kit (catalog# 5266645001, Roche) and counterstaining with Mayer’s hematoxylin. Quantitative analysis of the percentage of NGF-positive cells was performed using QuPath. Normality was analyzed using a Shapiro–Wilk test, and an unpaired *T*-test was performed for normally distributed data sets and a Mann–Whitney *U* test was performed for non-normal data sets.

## Results

**FIGURE 1:**
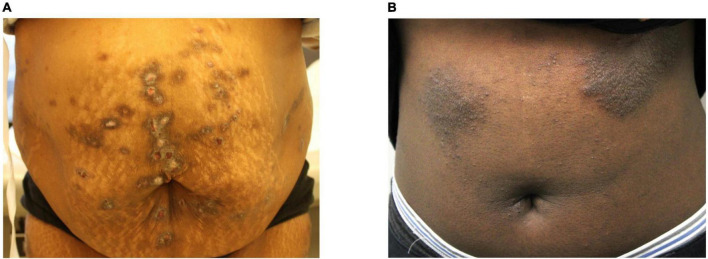
Clinical images of prurigo nodularis and atopic dermatitis lesions. **(A)** Prurigo nodularis patient with fibrotic and hyperkeratotic lesions on the abdomen. **(B)** Atopic dermatitis patient with lichenified lesions on the abdomen.

**FIGURE 2:**
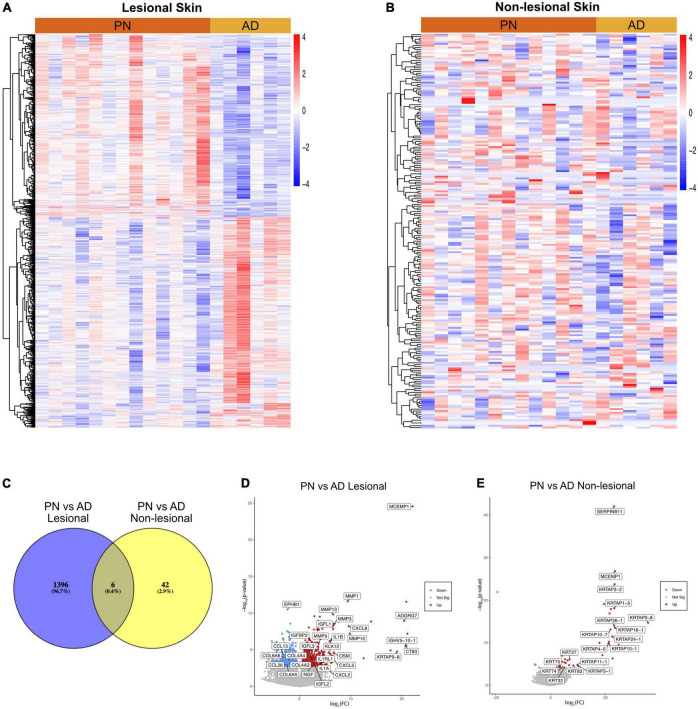
Transcriptomic comparisons of prurigo nodularis (PN) and atopic dermatitis (AD) skin. **(A)** Heatmap of gene expression for differentially expressed genes (DEGs) between PN lesional vs. AD lesional samples, where red is higher expression and blue is lower expression. **(B)** Heatmap of gene expression for differentially expressed genes (DEGs) between PN non-lesional vs. AD non-lesional samples, where red is higher expression and blue is lower expression. **(C)** Venn diagram of DEGs for PN lesional and AD lesional samples compared to PN non-lesional and AD non-lesional samples. **(D)** PN lesional vs. AD lesional volcano plot. **(E)** PN non-lesional vs. AD non-lesional volcano plot.

Transcriptome analysis was performed on a total of 38 lesional and non-lesional skin biopsies from 13 PN (mean age 54.8 ± 14.2 years, 84.6% female, and 76.9% African American) and 6 AD (mean age 55.2 ± 15.8 years, 83.3% female, and 100.0% African American) patients and was compared to the cutaneous transcriptome of 19 matched controls (Supplementary Table 1). Representative clinical pictures of PN and AD lesions are shown in Figure 1. RNA sequencing revealed 1,396 DEGs between lesional PN and AD skin (PN/AD L), 42 DEGs between non-lesional PN and AD skin (PN/AD NL), and 6 DEGs in common between PN/AD L and PN/AD NL (Figures 2A–C). The number of DEGs in the skin of PN and AD patients compared to controls are shown in Supplementary Figure 1. Comparing lesional PN skin to lesional AD skin and control skin, the significantly upregulated DEGs in PN included matrix metalloproteinases (MMP1, MMP3, MMP10, MMP13), OSM, NGF, IL1α, IL1β, CXCL2, CXCL8, and insulin-like growth factors (IGFL2, IGFL3) (Figure 2D and Supplementary Figure 1B). Importantly, these genes were not significantly upregulated in AD lesions compared to controls (Supplementary Figure 1C), indicating that these genes are uniquely upregulated in PN. Significantly downregulated DEGs in PN lesions compared to AD lesions included CCL13, CCL26, EPHB1, and collagens (COL4/6). Comparing non-lesional skin in PN and AD, PN skin showed significant upregulation of keratin-family genes (KRT/KRTAP) (Figure 2E). Similarly, these genes were not significantly upregulated in AD non-lesional skin compared to controls (Supplementary Figure 1F), affirming the unique expression of these genes in the non-lesional skin of PN.

**FIGURE 3:**
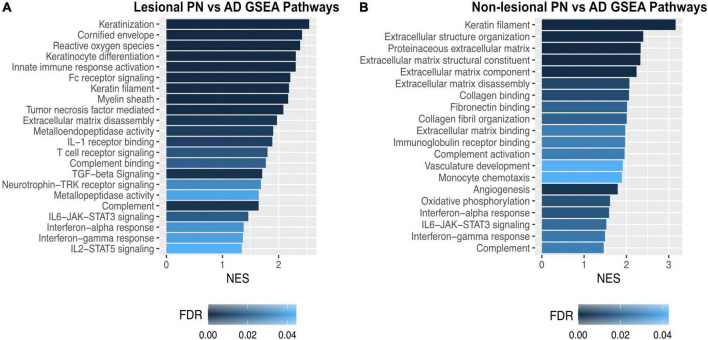
Gene set enrichment analysis of dysregulated genetic pathways in prurigo nodularis (PN) and atopic dermatitis (AD) skin. **(A)** Gene set enrichment analysis (GSEA) for selected significant immune pathways in PN lesional vs. AD lesional skin. **(B)** GSEA for selected significant immune pathways in PN non-lesional vs. AD non-lesional skin. FC, fold change; FDR, false discovery rate *p*-values; NES, normalized enrichment score.

Gene set enrichment analysis of lesional PN and AD skin revealed that PN lesions had higher enrichment of pathways including keratinization [normalized enrichment score (NES) 2.55, FDR < 10^–5^], cornified envelope (NES 2.41, FDR < 10^–5^), myelin sheath (NES 2.17, FDR 5.12 × 10^–4^), TGF-beta signaling (NES 2.09, FDR 0.001), extracellular matrix disassembly (NES 1.97, FDR 0.004), metalloendopeptidase activity (NES 1.90, FDR 0.008), and neurotrophin-TRK receptor signaling (NES 1.68, FDR 0.033) (Figure 3A). GSEA of non-lesional PN and AD skin revealed that PN had higher enrichment of pathways including keratin filament (NES 3.16, FDR < 10^–5^), extracellular structure organization (NES 3.40, FDR < 10^–5^), extracellular matrix disassembly (NES 2.07, FDR 0.01), and angiogenesis (NES 1.99, FDR 0.023) compared to AD (Figure 3B).

**FIGURE 4:**
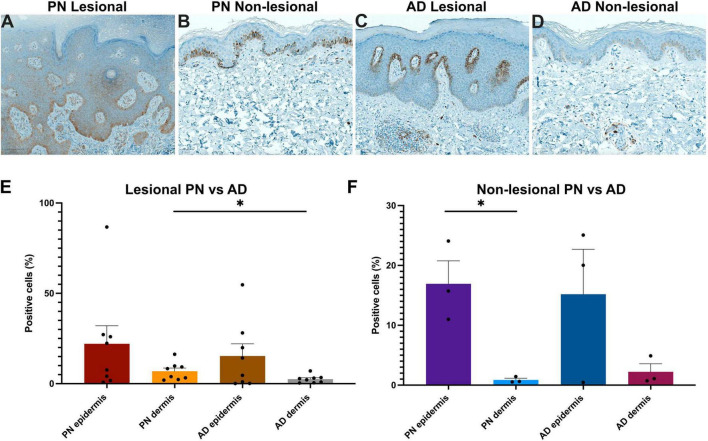
Immunohistochemistry (IHC) analysis of nerve growth factor (NGF) in prurigo nodularis (PN) and atopic dermatitis (AD) skin. **(A–D)** Representative NGF IHC staining in PN lesional, PN non-lesional, AD lesional, and AD non-lesional skin, respectively. Panels **(A,C)** 10×. Panels **(B,D)** 15×. **(E)** Quantification of the percentage of NGF-positive cells in lesional PN vs. lesional AD skin. **(F)** Quantification of the percentage of NGF-positive cells in non-lesional PN vs. non-lesional AD skin. *p < 0.05. The symbol “●” represents the percentage of positive cells for an individual sample.

Immunohistochemical quantification of NGF expression in matched PN and AD patients (Supplementary Table 2) corroborated the RNA sequencing findings of hyperkeratosis in PN lesions compared to AD lesions (Figures 4A–D) and further revealed that PN lesional samples had higher dermal NGF expression than AD lesional samples (6.89 vs. 2.51% positive cells, *p* = 0.038) (Figures 4E–F). In non-lesional PN samples, there was greater epidermal compared to dermal NGF expression (16.93 vs. 0.87% positive cells, *p* = 0.014).

## Discussion

This study revealed significant enrichment of extracellular matrix remodeling and neurotrophic signatures in PN compared to AD. NGF in the skin is crucial for the survival and regeneration of damaged cutaneous sensory nerves, and transcriptomic and immunohistochemical analysis demonstrated greater NGF expression in PN lesional dermal skin compared to AD. Prior studies have found decreased intraepidermal nerve fiber density, hypertrophy of papillary dermal nerves, altered Schwann cell morphology, and increased numbers of NGF-positive papillary dermal nerve fibers in PN skin compared to controls (10–15). These changes have been implicated in the development of chronic itch. While we found that NGF expression in the PN epidermis is comparable to that in AD epidermis, we additionally found that NGF upregulation is more pronounced in dermal PN than in dermal AD lesions. Transcriptomic analysis also revealed dysregulation in other neurotrophic modulators such as insulin-like growth factors (IGF/IGFL), IL-1β, and ephrin receptor B1 (EPHB1). These results suggest that PN patients experience greater degrees of cutaneous neural dysregulation compared to AD. This is consistent with prior reports that PN is strongly associated with peripheral neuropathies, and that treatments commonly used to treat neuropathic pain have been shown to benefit patients with PN, suggesting a role for neural dysregulation in its pathogenesis (16).

Furthermore, neural dysregulation in PN can be potentiated by alterations in the extracellular matrix. We found that PN lesions had decreased collagen VI and increased oncostatin M (OSM) and matrix metalloproteinases (MMPs) compared to AD lesions. Studies have shown that lack of collagen VI, which is necessary for maintaining nerve function and regeneration, can delay peripheral nerve regeneration (17). OSM, a cytokine with roles in proliferation or differentiation of hematopoietic and neuronal cells, can also modulate extracellular matrix components and maintain chronic inflammation (18, 19). These findings are concordant with human clinical trials to date demonstrating that OSM inhibition has greater efficacy in PN than in AD (20, 21). OSM can also upregulate MMP activity, which in turn enhances inflammation through degradation of extracellular structures, enabling immune cells to enter and exit skin, and activation of cytokines and chemokines (5, 22, 23). Immunomodulators activated by MMPs include IL-1β, CXCL5, and CXCL8, whose genes were upregulated in lesional PN skin and are implicated in inflammation and neuropathies (23–26). Alterations in extracellular matrix components can therefore be major contributors to enhanced inflammation, fibrosis, and neural dysregulation in PN.

Limitations of this study include sample size and patient recruitment from a single tertiary care center, and therefore our results are most applicable to this specific patient population. Nonetheless, we present novel findings demonstrating dysregulation of neural regeneration and extracellular matrix remodeling signatures in PN compared to AD patients, possibly explaining the morphological differences in their lesions. These findings provide deeper insight into the differences in pathogenesis between PN and AD and may aid in the identification of future therapeutic targets. Future studies are warranted to investigate the levels of neurotrophic factors and extracellular matrix dysregulation at different clinical stages of PN and in patients from different demographic populations.

## Data availability statement

The datasets presented in this article are not readily available because of ethical restrictions. Requests to access the datasets should be directed to the corresponding author.

## Ethics statement

The studies involving human participants were reviewed and approved by Johns Hopkins Institutional Review Board. The patients/participants provided their written informed consent to participate in this study.

## Author contributions

JD, VP, MM, MA, and SK contributed to conception and design of the study. JD, VP, MM, and CT organized the database. JD performed the statistical analysis and wrote the first draft of the manuscript. JD, VP, MM, and KL wrote sections of the manuscript. All authors contributed to manuscript revision and read and approved the submitted version.

## Supplementary material

Supplementary Figure 1 Transcriptomic comparisons of prurigo nodularis (PN) and atopic dermatitis (AD) to matched healthy control skin. **(A)** Venn diagram of DEGs for PN lesional vs. control samples, AD lesional vs. control samples, and PN lesional vs. AD lesional samples. **(B)** PN lesional vs. controls volcano plot. **(C)** AD lesional vs. controls volcano plot. **(D)** Venn diagram of DEGs for PN non-lesional vs. control samples, AD non-lesional vs. control samples, and PN non-lesional vs. AD non-lesional samples. **(E)** PN non-lesional vs. controls volcano plot. **(F)** AD non-lesional vs. controls volcano plot.

Supplementary Table 1 Study cohort demographic information for patients recruited for RNA sequencing analysis.

Supplementary Table 2 Study cohort demographic information for matched patients recruited to provide skin samples for immunohistochemistry analysis.

## References

[1] Williams K Huang A Belzberg M Kwatra S. Prurigo nodularis: pathogenesis and management. *J Am Acad Dermatol.* (2020) 83:1567–75. 10.1016/j.jaad.2020.04.182 32461078

[2] Boozalis E Tang O Patel S Semenov Y Pereira M Stander S Ethnic differences and comorbidities of 909 prurigo nodularis patients. *J Am Acad Dermatol.* (2018) 79:714–719.e3. 10.1016/j.jaad.2018.04.047 29733939 PMC6518410

[3] Wongvibulsin S Sutaria N Kannan S Alphonse M Belzberg M Williams K Transcriptomic analysis of atopic dermatitis in African Americans is characterized by Th2/Th17-centered cutaneous immune activation. *Sci Rep.* (2021) 11:11175. 10.1038/s41598-021-90105-w 34045476 PMC8160001

[4] Belzberg M Alphonse M Brown I Williams K Khanna R Ho B Prurigo nodularis is characterized by systemic and cutaneous T helper 22 immune polarization. *J Invest Dermatol.* (2021) 141:2208.e–18.e. 10.1016/j.jid.2021.02.749 33771530 PMC8384659

[5] Harper J Godwin H Green A Wilkes L Holden N Moffatt M A study of matrix metalloproteinase expression and activity in atopic dermatitis using a novel skin wash sampling assay for functional biomarker analysis. *Br J Dermatol.* (2010) 162:397–403. 10.1111/j.1365-2133.2009.09467.x 19804592

[6] Chen S Zhou Y Chen Y Gu J. Fastp: an ultra-fast all-in-one Fastq preprocessor. *Bioinformatics.* (2018) 34:i884–90. 10.1093/bioinformatics/bty560 30423086 PMC6129281

[7] Dobin A Davis C Schlesinger F Drenkow J Zaleski C Jha S Star: ultrafast universal RNA-Seq aligner. *Bioinformatics.* (2013) 29:15–21. 10.1093/bioinformatics/bts635 23104886 PMC3530905

[8] Liao Y Smyth G Shi W. Featurecounts: an efficient general purpose program for assigning sequence reads to genomic features. *Bioinformatics.* (2014) 30:923–30. 10.1093/bioinformatics/btt656 24227677

[9] Subramanian A Tamayo P Mootha V Mukherjee S Ebert B Gillette M Gene set enrichment analysis: a knowledge-based approach for interpreting genome-wide expression profiles. *Proc Natl Acad Sci USA.* (2005) 102:15545–50. 10.1073/pnas.0506580102 16199517 PMC1239896

[10] Andersen H Arendt-Nielsen L Gazerani P. Glial cells are involved in itch processing. *Acta Derm Venereol.* (2016) 96:723–7. 10.2340/00015555-2366 26864998

[11] Feuerman E Sandbank M. Prurigo nodularis. Histological and electron microscopical study. *Arch Dermatol.* (1975) 111:1472–7. 10.1001/archderm.111.11.1472 1200655

[12] Haas S Capellino S Phan N Böhm M Luger T Straub R Low density of sympathetic nerve fibers relative to substance P-positive nerve fibers in lesional skin of chronic pruritus and prurigo nodularis. *J Dermatol Sci.* (2010) 58:193–7. 10.1016/j.jdermsci.2010.03.020 20417061

[13] Harris B Harris K Penneys N. Demonstration by S-100 protein staining of increased numbers of nerves in the papillary dermis of patients with prurigo nodularis. *J Am Acad Dermatol.* (1992) 26:56–8. 10.1016/0190-9622(92)70006-2 1732336

[14] Pereira M Mühl S Pogatzki-Zahn E Agelopoulos K Ständer S. Intraepidermal nerve fiber density: diagnostic and therapeutic relevance in the management of chronic pruritus: a review. *Dermatol Ther (Heidelb).* (2016) 6:509–17. 10.1007/s13555-016-0146-1 27730494 PMC5120635

[15] Schuhknecht B Marziniak M Wissel A Phan N Pappai D Dangelmaier J Reduced intraepidermal nerve fibre density in lesional and nonlesional prurigo nodularis skin as a potential sign of subclinical cutaneous neuropathy. *Br J Dermatol.* (2011) 165:85–91. 10.1111/j.1365-2133.2011.10306.x 21410670

[16] Hughes J Woo T Belzberg M Khanna R Williams K Kwatra M Association between prurigo nodularis and etiologies of peripheral neuropathy: suggesting a role for neural dysregulation in pathogenesis. *Medicines (Basel).* (2020) 7:4. 10.3390/medicines7010004 31936197 PMC7167799

[17] Chen P Cescon M Zuccolotto G Nobbio L Colombelli C Filaferro M Collagen Vi regulates peripheral nerve regeneration by modulating macrophage recruitment and polarization. *Acta Neuropathol.* (2015) 129:97–113. 10.1007/s00401-014-1369-9 25421425

[18] Gearing D Ziegler S Comeau M Friend D Thoma B Cosman D Proliferative responses and binding properties of hematopoietic cells transfected with low-affinity receptors for leukemia inhibitory factor, oncostatin M, and ciliary neurotrophic factor. *Proc Natl Acad Sci USA.* (1994) 91:1119–23. 10.1073/pnas.91.3.1119 8302840 PMC521465

[19] Ryan R Martin B Mellor L Jacob R Tawara K McDougal O Oncostatin M binds to extracellular matrix in a bioactive conformation: implications for inflammation and metastasis. *Cytokine.* (2015) 72:71–85. 10.1016/j.cyto.2014.11.007 25622278 PMC4328881

[20] Kiniksa Pharmaceuticals Ltd. *Kiniksa Announces Interim Data from Kpl-716 Repeated-Single-Dose Phase 1b Clinical Trial.* Los Angeles, CA: GlobeNewswire, Inc (2019).

[21] Kiniksa Pharmaceuticals Ltd. *Kiniksa Announces Phase 2 Clinical Trial of Vixarelimab (Kpl-716) in Prurigo Nodularis Meets Primary Efficacy Endpoint.* Los Angeles, CA: GlobeNewswire, Inc (2020).

[22] O’Kane C Elkington P Friedland J. Monocyte-dependent oncostatin M and Tnf-alpha synergize to stimulate unopposed matrix metalloproteinase-1/3 secretion from human lung fibroblasts in tuberculosis. *Eur J Immunol.* (2008) 38:1321–30. 10.1002/eji.200737855 18398932

[23] Parks W Wilson C López-Boado Y. Matrix metalloproteinases as modulators of inflammation and innate immunity. *Nat Rev Immunol.* (2004) 4:617–29. 10.1038/nri1418 15286728

[24] Abbadie C. Chemokines, chemokine receptors and pain. *Trends Immunol.* (2005) 26:529–34. 10.1016/j.it.2005.08.001 16099720

[25] Trier A Mack M Kim B. The neuroimmune axis in skin sensation, inflammation, and immunity. *J Immunol.* (2019) 202:2829–35. 10.4049/jimmunol.1801473 31061146 PMC6563610

[26] Xu W Zhu M Yuan S Yu W. Spinal Cxcl5 contributes to nerve injury-induced neuropathic pain via modulating gsk-3β phosphorylation and activity in rats. *Neurosci Lett.* (2016) 634:52–9. 10.1016/j.neulet.2016.10.004 27717828

