# Supplementary figures and images for: Extracellular matrix and dermal nerve growth factor dysregulation in prurigo nodularis compared to atopic dermatitis
Source: Front Med (Lausanne). 2022 Dec 21;9:1022889. doi: 10.3389/fmed.2022.1022889 (PMC9810753; doi:10.3389/fmed.2022.1022889)

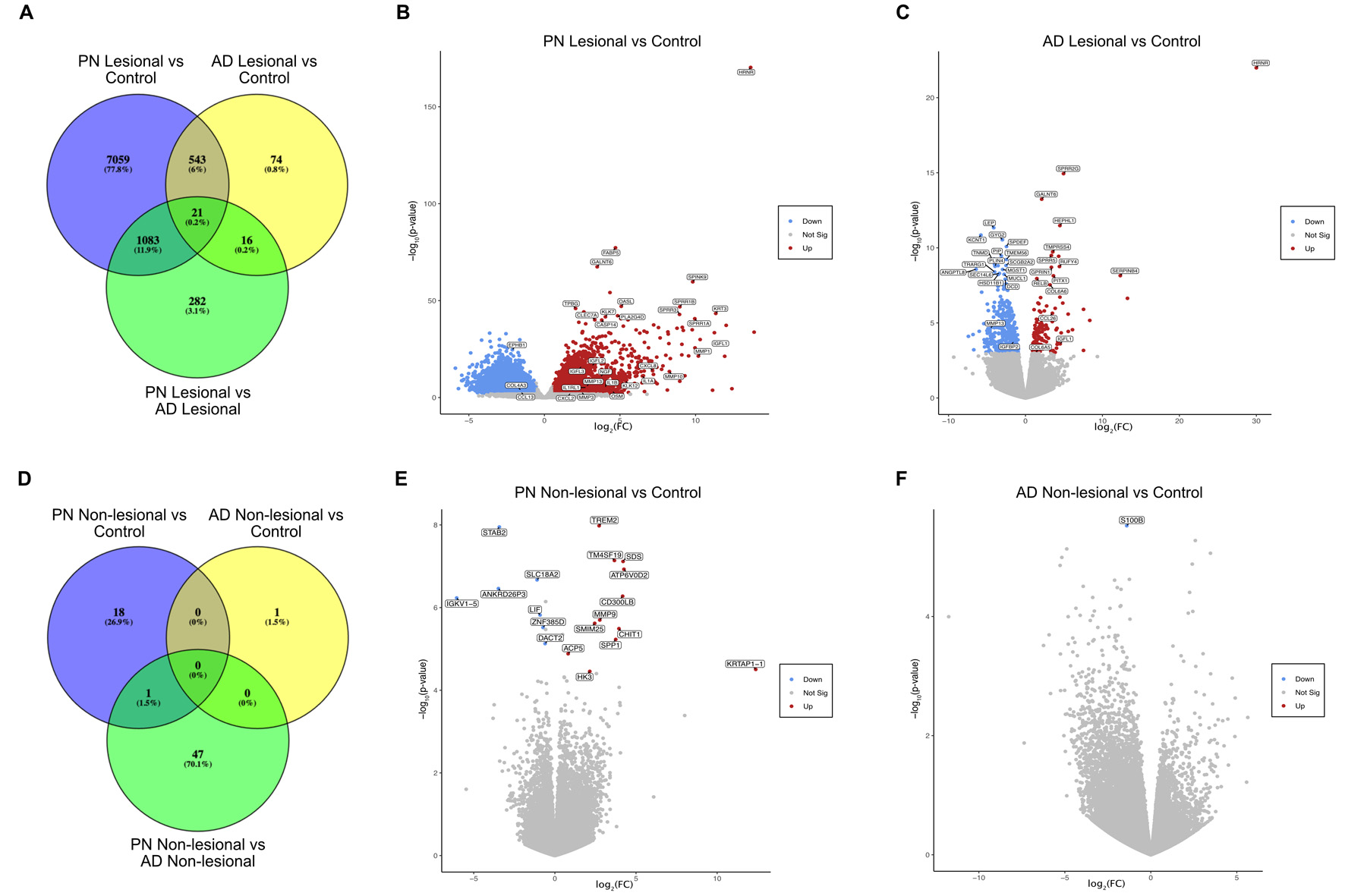

Supplement: Supplementary Figure 1 — Transcriptomic comparisons of prurigo nodularis (PN) and atopic dermatitis (AD) to matched healthy control skin. (A) Venn diagram of DEGs for PN lesional vs. control samples, AD lesional vs. control samples, and PN lesional vs. AD lesional samples. (B) PN lesional vs. controls volcano plot. (C) AD lesional vs. controls volcano plot. (D) Venn diagram of DEGs for PN non-lesional vs. control samples, AD non-lesional vs. control samples, and PN non-lesional vs. AD non-lesional samples. (E) PN non-lesional vs. controls volcano plot. (F) AD non-lesional vs. controls volcano plot. [file Image_1.JPEG]
