# Supplementary material for: Extracellular matrix and dermal nerve growth factor dysregulation in prurigo nodularis compared to atopic dermatitis
Source: Front Med (Lausanne). 2022 Dec 21;9:1022889. doi: 10.3389/fmed.2022.1022889 (PMC9810753; doi:10.3389/fmed.2022.1022889)
Supplement: Supplementary Table 1 — Study cohort demographic information for patients recruited for RNA sequencing analysis. [file Table_1.DOCX]

**Supplementary Table S1.** Study cohort demographic information for patients recruited for RNA sequencing analysis

| Patient | Age | Sex | Race | Specimen Site | Itch NRS | Healthy Control | Age | Sex | Race | Specimen Site |
| --- | --- | --- | --- | --- | --- | --- | --- | --- | --- | --- |
| 1 PN | 59 | F | White | Shin | Moderate-Severe | **1 HC** | 58 | F | White | Shin |
| 2 PN | 49 | F | AA | Arm | Severe | **2 HC** | 53 | F | AA | Arm |
| 3 PN | 74 | F | White | Back | Severe | **3 HC** | 75 | F | White | Back |
| 4 PN | 27 | F | AA | Back | Severe | **4 HC** | 28 | F | AA | Back |
| 5 PN | 68 | F | White | Leg | Severe | **5 HC** | 65 | F | White | Leg |
| 6 PN | 57 | F | AA | Leg | Severe | **6 HC** | 58 | F | AA | Leg |
| 7 PN | 68 | F | AA | Leg | Severe | **7 HC** | 58 | F | AA | Leg |
| 8 PN | 34 | F | AA | Thigh | Severe | **8 HC** | 29 | F | AA | Leg |
| 9 PN | 65 | F | AA | Thigh | Moderate-Severe | **9 HC** | 63 | F | AA | Leg |
| 10 PN | 57 | M | AA | Arm | Severe | **10 HC** | 50 | M | AA | Arm |
| 11 PN | 64 | F | AA | Arm | Severe | **11 HC** | 66 | F | AA | Arm |
| 12 PN | 39 | F | AA | Arm | Severe | **12 HC** | 38 | F | AA | Arm |
| 13 PN | 51 | M | AA | Back | Severe | **13 HC** | 53 | M | AA | Back |
| 1 AD | 23 | F | AA | Arm | Severe | **14 HC** | 29 | F | AA | Arm |
| 2 AD | 62 | M | AA | Chest | Severe | **15 HC** | 53 | M | AA | Chest |
| 3 AD | 61 | F | AA | Leg | Severe | **16 HC** | 66 | F | AA | Leg |
| 4 AD | 62 | F | AA | Back | Severe | **17 HC** | 63 | F | AA | Back |
| 5 AD | 62 | F | AA | Leg | Severe | **18 HC** | 58 | F | AA | Leg |
| 6 AD | 61 | F | AA | Leg | Moderate-Severe | **19 HC** | 66 | F | AA | Leg |

Abbreviations: PN, prurigo nodularis; AD, atopic dermatitis; HC, healthy control; AA, African American; F, female; M, male; NRS, numeric rating scale.
