# Supplementary material for: Extracellular matrix and dermal nerve growth factor dysregulation in prurigo nodularis compared to atopic dermatitis
Source: Front Med (Lausanne). 2022 Dec 21;9:1022889. doi: 10.3389/fmed.2022.1022889 (PMC9810753; doi:10.3389/fmed.2022.1022889)
Supplement: Supplementary Table 2 — Study cohort demographic information for matched patients recruited to provide skin samples for immunohistochemistry analysis. [file Table_2.DOCX]

**Supplementary Table S2.** Study cohort demographic information for matched patients recruited to provide skin samples for immunohistochemistry analysis

| Comparison | Patient with PN | Age | Sex | Race | Patient with AD | Age | Sex | Race |
| --- | --- | --- | --- | --- | --- | --- | --- | --- |
| Lesional Skin | 1 PN | 59 | F | White | 7 AD | 60 | F | White |
| Lesional Skin | 14 PN | 61 | M | AA | 2 AD | 62 | M | AA |
| Lesional Skin | 4 PN | 27 | F | AA | 1 AD | 23 | F | AA |
| Lesional Skin | 8 PN | 34 | F | AA | 8 AD | 24 | F | AA |
| Lesional Skin | 9 PN | 65 | F | AA | 5 AD | 62 | F | AA |
| Lesional Skin | 15 PN | 57 | F | AA | 6 AD | 61 | F | AA |
| Lesional Skin | 16 PN | 68 | F | AA | 9 AD | 69 | F | AA |
| Lesional Skin | 17 PN | 75 | F | AA | 10 AD | 71 | F | AA |
| Non-lesional Skin | 4 PN | 27 | F | AA | 1 AD | 23 | F | AA |
| Non-lesional Skin | 9 PN | 65 | F | AA | 6 AD | 61 | F | AA |
| Non-lesional Skin | 16 PN | 68 | F | AA | 5 AD | 62 | F | AA |

Abbreviations: PN, prurigo nodularis; AD, atopic dermatitis; AA, African American; F, female; M, male.
